# SUMOylation regulates ciliary localization of olfactory signaling proteins

**DOI:** 10.1242/jcs.164673

**Published:** 2015-05-15

**Authors:** Jeremy C. McIntyre, Ariell M. Joiner, Lian Zhang, Jorge Iñiguez-Lluhí, Jeffrey R. Martens

**Affiliations:** 1 Department of Pharmacology and Therapeutics, University of Florida, PO Box 100267, Gainesville, FL 32610, USA; 2 Department of Pharmacology, University of Michigan, Ann Arbor, MI 48109, USA

**Keywords:** Cilia, Protein Trafficking, SUMO

## Abstract

Cilia are evolutionarily conserved organelles found on many mammalian cell types, including neuronal populations. Although neuronal cilia, including those on olfactory sensory neurons (OSNs), are often delineated by localization of adenylyl cyclase 3 (AC3, also known as ADCY3), the mechanisms responsible for targeting integral membrane proteins are largely unknown. Post-translational modification by small ubiquitin-like modifier (SUMO) proteins plays an important role in protein localization processes such as nuclear–cytosolic transport. Here, we identified through bioinformatic analysis that adenylyl cyclases harbor conserved SUMOylation motifs, and show that AC3 is a substrate for SUMO modification. Functionally, overexpression of the SUMO protease SENP2 prevented ciliary localization of AC3, without affecting ciliation or cilia maintenance. Furthermore, AC3-SUMO mutants did not localize to cilia. To test whether SUMOylation is sufficient for cilia entry, we compared localization of ANO2, which possesses a SUMO motif, and ANO1, which lacks SUMOylation sites and does not localize to cilia. Introduction of SUMOylation sites into ANO1 was not sufficient for ciliary entry. These data suggest that SUMOylation is necessary but not sufficient for ciliary trafficking of select constituents, further establishing the link between ciliary and nuclear import.

## INTRODUCTION

Cilia are evolutionarily conserved organelles projecting from the surface of cells and are important for motility, sensing external stimuli and maintaining cellular homeostasis. Defects in cilia formation and function underlie a growing number of human diseases that affect numerous organ systems. In the olfactory system, the multiple cilia that extend from the dendritic knobs of olfactory sensory neurons (OSNs) are essential for function given that ciliary disruption leads to anosmia (Kulaga et al., 2004; McEwen et al., 2007; McIntyre et al., 2012). Functional odorant detection depends on a ciliary-localized signaling pathway involving odorant receptors coupled to adenylyl cyclase 3 (AC3, also known as ADCY3) (Bakalyar and Reed, 1990; Bönigk et al., 1999; Buck and Axel, 1991; Feinstein et al., 2004; Menco et al., 1997; Schwarzenbacher et al., 2005; Stephan et al., 2009; Wong et al., 2000). The ciliary localization of AC3 is also characteristic of primary neurons where it is enriched in the primary cilia projecting from neurons throughout many regions of the brain (Berbari et al., 2007; Bishop et al., 2007). Although localization of AC3 to cilia in both of these systems is necessary for proper function, little is known about the mechanisms that are responsible for its targeting (Guadiana et al., 2013; Kaneko-Goto et al., 2013; Wang et al., 2011; Wong et al., 2000).

For ciliary localization of transmembrane proteins, mechanisms relying on the use of specific signal sequences such as the RVxP motif and the Ax[S/A]xQ motif have been identified (Berbari et al., 2008a; Dishinger et al., 2010; Geng et al., 2006). For example, the Ax[S/A]xQ motif and interactions with chaperone proteins regulates the ciliary localization of several G-protein-coupled receptors (GPCRs) (Berbari et al., 2008a; Berbari et al., 2008b; Sun et al., 2012). In the OSNs, ciliary trafficking of olfactory cyclic-nucleotide-gated (CNG) channels requires formation of a heteromeric complex between CNGA2 and the CNGB1b subunit, which contains a RVxP motif (Jenkins et al., 2006; Michalakis et al., 2006). The proper context is essential, however, because insertion of this motif into CNGA2 is not sufficient for trafficking of the CNGA2 subunit on its own (Jenkins et al., 2006). Notably, CNG channel trafficking also depends on phosphorylation of CNGB1 by the serine/threonine protein kinase casein kinase II (CK2) and interaction with phosphofurin acidic cluster-sorting protein 1 (PACS-1) (Jenkins et al., 2009). Although these studies have identified mechanisms involved in ciliary CNG trafficking, these mechanisms do not account for the ciliary enrichment of other olfactory signaling proteins. For example, overexpression of a dominant-negative PACS-1 protein in OSNs prevents CNGA2 localization, but does not affect ciliary localization of AC3 (Jenkins et al., 2009). Retention of selective ciliary entry mechanisms has also been demonstrated in OSNs of *Cep290* and *Cetn2* mutant mice where ciliary transition zone and basal body function is disrupted (McEwen et al., 2007; Ying, 2014). Together these findings argue that ciliary targeting mechanisms are protein specific and likely involve multiple processes with some proteins requiring several different mechanisms. To fully elucidate the mechanisms regulating ciliary entry, comparisons to other selective entry compartments might provide additional insight.

Mechanisms that are known to regulate nuclear import have recently been shown to function in ciliary import. Similar to in the nucleus, molecular size and nucleoporins regulate the ability of soluble proteins to diffuse into the cilium (Breslow et al., 2013; Kee et al., 2012; Kee and Verhey, 2013; Lin et al., 2013). In addition, basic sequences similar to nuclear localization signals have been identified in several ciliary proteins and these sequences regulate localization into the cilium (Dishinger et al., 2010; Hurd et al., 2011). Finally, nuclear import of many proteins involves a Ran GTPase gradient and chaperone proteins such as importin-β2 (also known as TNPO1). These same mechanisms have also been implicated in both ciliogenesis and trafficking of proteins in the cilium (Dishinger et al., 2010; Fan et al., 2011; Hurd et al., 2011; Maiuri et al., 2013).

Given the identified similarities between nuclear and ciliary entry, it is reasonable that other mechanisms known to regulate protein localization into the nucleus also act at the cilium. In this regard, the post-translational modification of proteins through the reversible covalent conjugation of small-ubiquitin like modifier (SUMO) proteins plays important roles in nucleocytoplasmic transport. In particular, the localization of RanGAP1 to the cytoplasmic face of nuclear pore filaments is essential for establishment of the Ran GTPase gradient and depends on RanGap SUMOylation as well as on the interaction between the nucleoporin Nup358 (also known as RANBP2) and the SUMO moiety on RanGap (Hutten et al., 2008; Matunis et al., 1996). In addition, the SUMOylation status of multiple proteins regulates their nuclear or cytoplasmic localization mainly by altering their interactions with other proteins (Hutten et al., 2008; Klein and Nigg, 2009; Majumdar et al., 2011; Pichler and Melchior, 2002; Zhang et al., 2002). Notably, SUMOylation is not restricted to soluble proteins but also plays important roles in the function and subcellular trafficking of integral membrane proteins. For example, the functional properties of several K^+^ channels are altered by SUMO modification (Benson et al., 2007; Plant et al., 2010; Plant et al., 2011; Qi et al., 2014). Interestingly, SUMOylation also regulates the trafficking and surface expression of a subset of transmembrane proteins such as the membrane insertion of the GluA1 AMPAR subunit (also known as GRIA1) and the activity dependent increase in AMPAR surface expression (Jaafari et al., 2013). These observations led us to consider that the conjugation of SUMO to polytopic membrane proteins could influence their ciliary localization.

Here, we demonstrate a new and direct role of SUMOylation in the ciliary localization of the adenylyl cyclase isoform AC3. The results from this study demonstrate that AC3 is a substrate for SUMOylation and that this modification is found on endogenous AC3 from olfactory cilia. Altering the SUMOylation status of AC3, either through overexpression of SUMO peptidases or mutation of the SUMO acceptor site within the protein, inhibits ciliary localization. Using the phylogenetically related integral membrane proteins, annoctamin 1 (ANO1) and annoctamin 2 (ANO2) which differ in their ciliary localization and possession of SUMOylation motifs, we also show that introduction of SUMOylation sites is not sufficient to drive entry into the cilium. These results are the first to show that SUMO modification of transmembrane proteins regulates their enrichment in the primary cilium and further highlight the similarities in the mechanisms used for nuclear and ciliary entry.

## RESULTS

### Adenylyl cyclase 3 contains a conserved consensus SUMOylation motif and is a SUMO substrate *in vitro* and *in vivo*

**Fig. 1.**
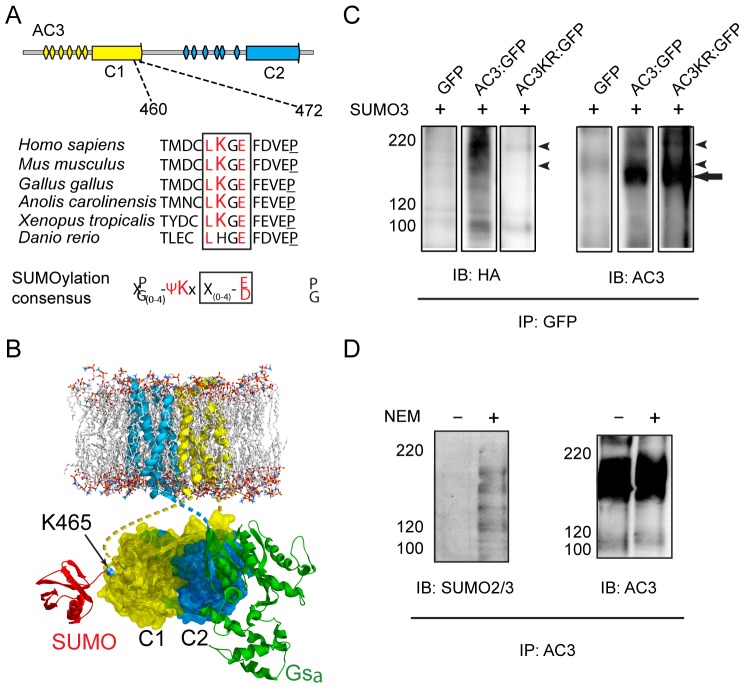
**AC3 harbors conserved SUMOylation motifs and is modified by SUMOylation *in vitro* and *in vivo.*** (A) Diagram of AC3 protein structure with the 12 ransmembrane domains, and two catalytic domains highlighted. SUMOylation occurs at the consensus sequence Ψ-K-X-D/E (Ψ is any large hydrophobic residue), with SUMO being conjugated to the lysine residue. SUMOsp 2.0 software revealed the presence of predicted SUMOylation sites in AC3 proteins from multiple species. (B) Proposed model of AC3, with a single conjugated SUMO protein. This model predicts that interaction of AC3 with SUMO would not necessarily interfere with Gαs binding. (C). Immunoprecipitation experiments from HEK293 cells co-transfected with GFP, AC3–GFP (AC3:GFP), or AC3K465R–GFP (AC3K465R:GFP), and HA–SUMO3 and Ubc9. Immunoprecipitation was performed with rabbit anti-GFP and immunoblotted (IB) with mouse anti-HA antibody (left panel). The membrane was then stripped and blotted with rabbit anti-AC3 antibody (right panel). Arrowheads denote SUMO-modified AC3 bands. The arrow marks the major AC3 band. (D) Endogenous AC3 is SUMOylated. Whole olfactory epithelium was homogenized either in the absence (–) or presence (+) of N-ethylmaleimide (NEM). Homogenates were pre-cleared with Protein A beads, and immunoprecipated with rabbit anti-AC3 antibody. Samples were probed with a monoclonal antibody against SUMO2 and SUMO3 (left panel), which only revealed bands in the NEM-treated sample. Probing for AC3 (right panel) shows similar efficiencies in immunoprecipitation. Numbers to the left of blots indicate molecular mass (kDa).

Given the established role for SUMO modifications in regulation of both membrane and nuclear trafficking, we sought to investigate a potential role for SUMOylation in regulating ciliary localization of AC3. Bioinformatic analysis of AC3 identified the presence of the consensus SUMOylation motif Ψ-K-X-D/E (Ψ is any large, hydrophobic residue), centered on K465 in the predicted third intracellular loop of the protein (Fig. 1A) (Ren et al., 2009). Modeling of SUMO3 in conjunction with the sequence of AC3 reveals that binding of SUMO3 would occur in a manner that would not inhibit interaction of AC3 with the stimulatory G protein (Fig. 1B). This sequence is highly conserved across vertebrate species; however, the specific motif is absent from the zebrafish (*Danio rerio*) sequence, although putative SUMO motifs are present at other positions in the full sequence. AC3 belongs to the type III family of adenylyl cyclase proteins, which consist of ten distinct isoforms. Interestingly, in addition to AC3, several other members are also reported to localize to cilia, including AC2, AC4, AC6, AC5 and AC8 (Choi et al., 2011; Masyuk et al., 2008; Wong et al., 2000). Analysis of all ten family members from the mouse genome reveals at least one predicted SUMO site in each sequence, suggesting that SUMOylation of transmembrane adenylate cyclase proteins is important for their trafficking and/or function.

The presence of putative modification sites indicates that AC3 is a likely target of SUMO modification. To evaluate whether AC3 is modified by SUMOylation in live cells, we immunoprecipitated GFP-tagged AC3 from HEK293 cells co-expressing HA–SUMO3 and Ubc9, the E2 SUMO-conjugating enzyme. Our choice of SUMO3 over the two other SUMO isoforms (SUMO1 and SUMO2) is based on its higher accumulation on modified proteins (Mukherjee et al., 2009) and its greater cytoplasmic localization and thus availability for conjugation to plasma membrane proteins (Ayaydin and Dasso, 2004; Benson et al., 2007). Western blot analysis of the samples using an anti-HA antibody revealed that HA-immunoreactive bands can be detected in samples expressing AC3–GFP but not in those derived from cells expressing HA–SUMO3 and GFP (Fig. 1C, left). Comparison to samples probed with an anti-AC3 antibody indicated that the HA immunoreactive species migrate with an apparent molecular mass that is greater than the main AC3–GFP form but coincided with minor AC3 immunoreactive species (Fig. 1C, right). The minor intensity of these bands is consistent with the relatively low steady state of SUMO modification of most proteins (Benson et al., 2007; Johnson, 2004). Notably, analysis of samples from cells expressing an AC3-SUMO mutant showed that the upper HA-immunoreactive band was not present, further indicating a specificity of SUMO binding to K465. These data demonstrate that AC3 can be modified by SUMOylation in a heterologous system. To test whether endogenous AC3 is a SUMO substrate, olfactory mucosa was isolated from wild-type mice in either the presence or absence of N-ethylmaleimide (NEM), a SUMO-specific deconjugating enzyme inhibitor (Benson et al., 2007). Immunoprecipitation of AC3 was performed with a rabbit anti-AC3 antibody from the tissue lysate. Blotting for endogenous SUMO2 and SUMO3, we were able to detect high molecular mass bands in samples that had been treated with NEM (Fig. 1D). These bands were not present in the samples prepared in the absence of NEM, indicating that SUMO2 and SUMO3 were interacting with AC3. As endogenous AC3 from the olfactory epithelium is highly glycosylated, the native band runs higher than the predicted molecular mass. In the olfactory epithelium samples several AC3 bands that had been modified with SUMO2 or SUMO3 appear to be closer in size to the glycosylated protein as the shift in molecular mass is not as great. Additionally several SUMO bands were also identified running between 120 and 150 kDa. These bands might correspond to modifications of the native AC3 proteins, which has a molecular mass of ∼120 kDa.

### SUMO isoforms differentially associate with cilia

**Fig. 2.**
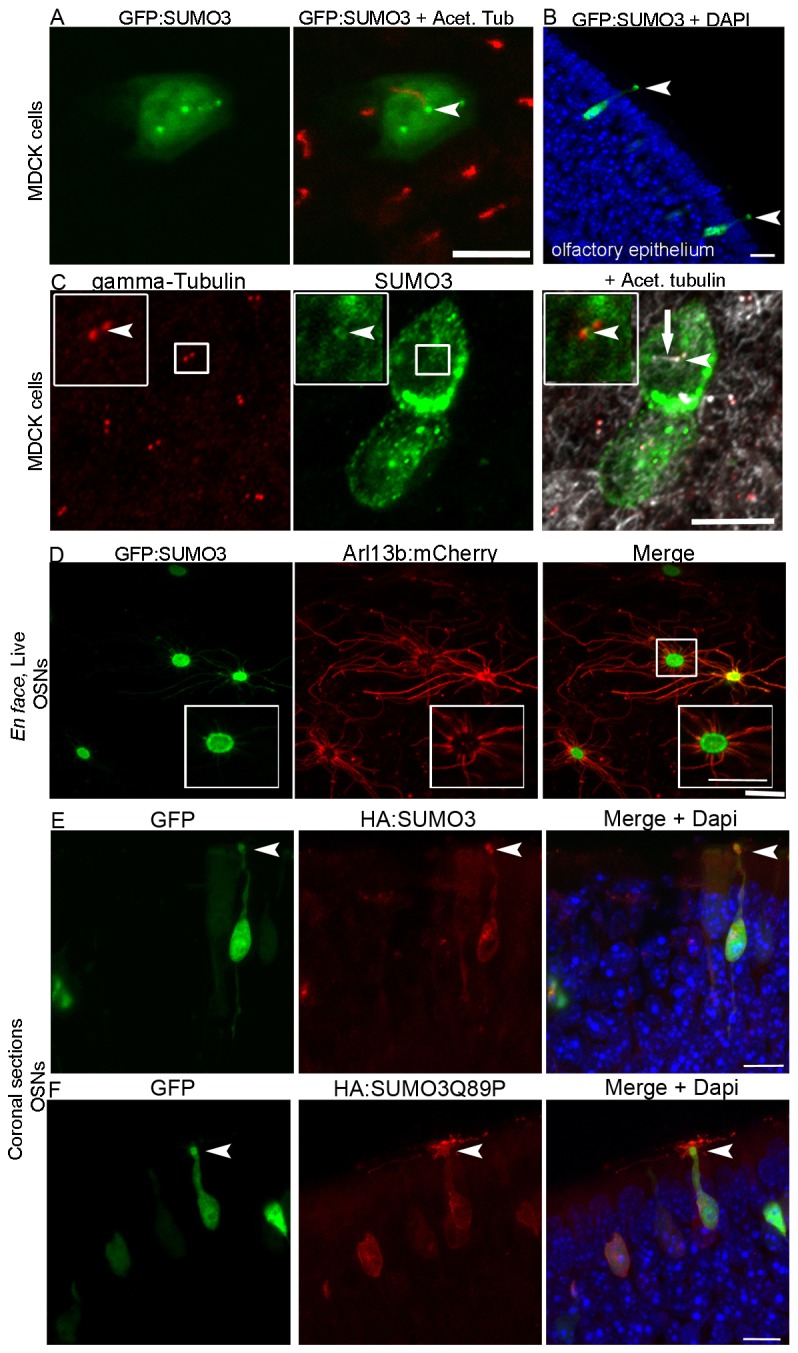
**Proteins involved in the SUMOylation pathway associate with cilia.** (A) Transfection of MDCK cells with GFP–SUMO (GFP:SUMO3), shows accumulation of SUMO3 at the base of cilia, marked by acetylated α-tubulin. (B) Immunostaining for the basal body protein, γ-tubulin corroborates the enrichment at the base of the cilium. (C) Ectopic expression of GFP–SUMO3 in OSNs reveals enrichment in dendritic knobs and cell bodies in fixed coronal sections of olfactory epithelium. (D) *En face* images of OSN knobs transduced with GFP–SUMO3 and Arl13b–mCherry (Arl13b:mCherry). The GFP signal is strongest in the knob, but can be seen in the cilia. Inset shows an image from a 1-µm-thick section, in which it is evident that the GFP–SUMO3 signal is strongest as a ring around the knob, at the base of the projecting cilia. (E) When expressed in OSNs by adenovirus transduction, HA–SUMO3 (HA:SUMO3) is enriched in the nucleus and dendritic knobs, with the HA signal not apparent in the cilia layer. (F) Non-cleavable HA–SUMO3-Q89P (HA:SUMO3Q89P), however localizes to olfactory cilia. OSNs were co-transduced with GFP adenovirus to label transduced neurons as a control. Scale bars: 10 μm.

Our results provide strong evidence that AC3 is covalently modified by SUMO both *in vivo* and *in vitro.* However, because AC3 also localizes to other regions of the cell, we sought to determine whether participants in the SUMOylation pathway associate with the cilium. In mammals, three SUMO isoforms competent for conjugation have been identified, of which SUMO3 expression is considered more cytoplasmic. To determine whether the extra-nuclear expression of this isoform localizes to cilia we expressed GFP-tagged SUMO3 in both cultured cells and *in vivo* in native OSNs. In ciliated MDCKII cells, GFP–SUMO3 was found throughout the cell cytoplasm; however it also accumulated at the base of cilia labeled with acetylated α-tubulin (Fig. 2A). The SUMO3 signal also colocalized with γ-tubulin, a marker for the basal bodies of cilia (Fig. 2C). In contrast, SUMO1 and SUMO2 were found to be mostly localized to the nucleus and not enriched at the base of cilia (supplementary material Fig. S1). To test whether GFP–SUMO3 localization to the ciliary base occurred in native OSNs, wild-type mice were intranasally injected with a GFP–SUMO3 adenovirus. After waiting 10 days to allow for virus expression, animals were killed and coronal sections were made through the olfactory epithelium. In OSNs, GFP–SUMO3 localized throughout the cell body and nucleus, and appeared to be enriched at the base of cilia in the dendritic knobs, but was not detected in the cilia layer, found along the apical surface of the olfactory epithelium (Fig. 2B). To further localize GFP–SUMO3 in OSNs and avoid possible fixation artifacts we used live *en face* imaging, which allows for a top down perspective of the dendritic knobs (McIntyre et al., 2012; Williams et al., 2014). Wild-type mice were injected with GFP–SUMO3 and Arl13b–mCherry, to effectively label the cilia projecting from each knob. In *en face* images, GFP–SUMO3 is strongest in the knobs, with weak signals in the cilia, seen by minimal colocalization with Arl13b–mCherry (Fig. 2D). However, some signal is obvious, suggesting that it is capable of entering. As the dendritic knobs possess the basal bodies for each cilium, we analyzed 1-µm-thick images. In these it is evident that the GFP–SUMO3 signal is strongest as a ring around the knob, adjacent to projections of Arl13b–mCherry cilia (Fig. 2D, inset). Given our results demonstrating that AC3 is a substrate for SUMO3 modification, the lack of robustly detectable SUMO3 in the primary cilium was surprising. A possible explanation for this is that the SUMO modification is transient and combined with a potentially limited fraction of ciliated proteins that are SUMO modified, the modification is difficult to detect under native conditions. Therefore to enrich the pool of ciliary proteins that are modified by SUMOylation we used a non-cleavable SUMO protein mutant, SUMO3-Q89P that is resistant to removal mediated by the SENP family of peptidases once conjugated to a target protein (Mukherjee et al., 2009). Wild-type SUMO3 and SUMO3-Q89P with N-terminal HA tags were then expressed in OSNs by adenoviral transduction. Animals were co-injected with a GFP-expressing adenovirus as a secondary label for transduced OSNs. Similar to GFP–SUMO3, wild-type HA–SUMO3 was localized to the dendritic knobs and cell bodies but absent from cilia (Fig. 2E). In contrast, whereas HA–SUMO3-Q89P localizes to the cell body and dendrites, it was also detected in the cilia projecting from OSNs (Fig. 2F). These data demonstrate that SUMO3 associates with the cilia of multiple cell types. Furthermore, the non-cleavable SUMO mutant data suggests that endogenous SUMO-modified proteins can exist within cilia of native cells *in vivo*.

### Proteolytic cleavage of SUMO alters ciliary localization AC3

**Fig. 3.**
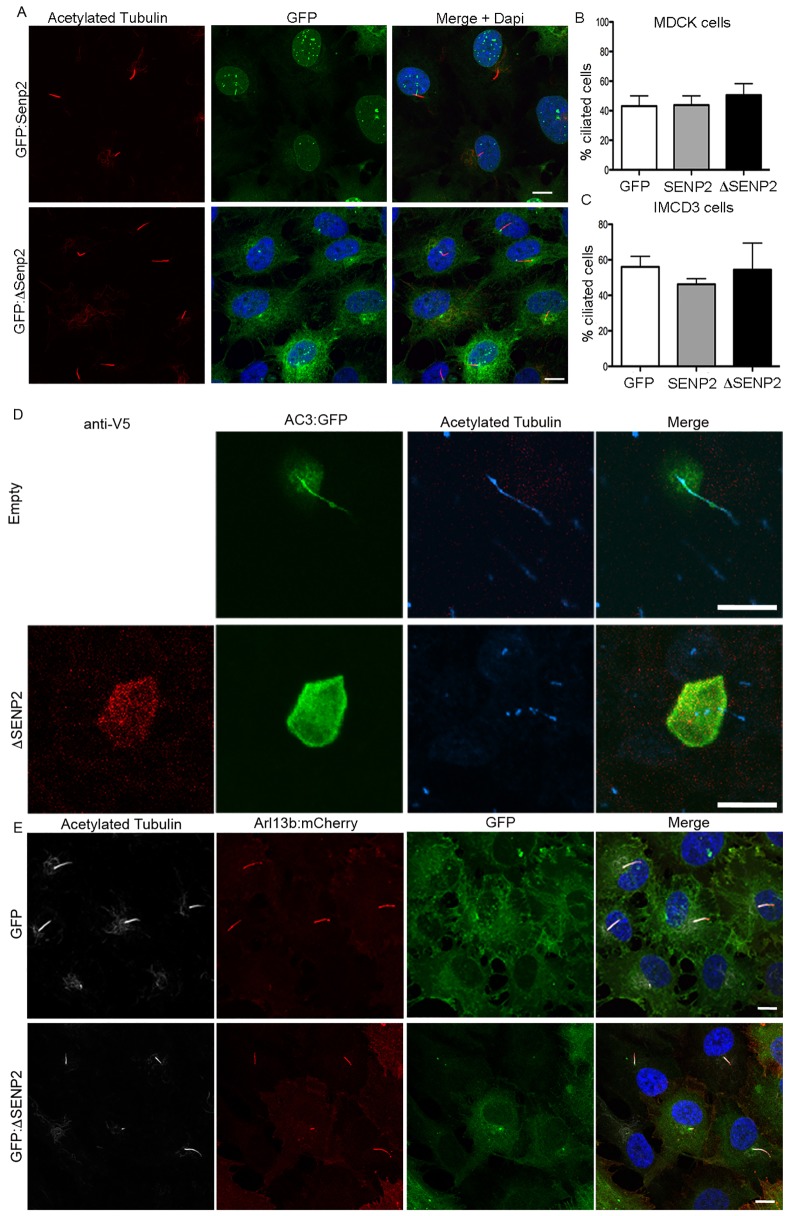
**Overexpression of ΔSENP2 blocks ciliary localization.** (A) Transduction of IMCD3 cells with GFP–SENP2 (GFP:SENP2, top) reveals SENP2 localization to the nuclear membrane, whereas expression of a N-terminus truncation (amino acids 1–82) of SENP2 (GFP:ΔSENP2; bottom) results in accumulation at the base of cilia. Neither SENP2 nor ΔSENP2 alters the presence of cilia as seen by immunostaining for acetylated α-tubulin in IMCD3 cells. (B,C) Quantification of the percentage of ciliated cells following transfection with either GFP, GFP–SENP2, or GFP–ΔSENP2 in both IMCD3 (B) or MDCKII (C) cells. Results are mean±s.e.m. (*n*=3 replicates per treatment). (D) AC3–GFP (AC3:GFP) localizes to cilia of MDCK cells as seen by colocalization with acetylated α-tubulin. Co-expression of AC3–GFP with ΔSENP2 prevents AC3–GFP localization in cilia. (E) Co-expression of GFP–ΔSENP2 with Arl13b–mCherry does not alter its entry to the cilium as seen by colocalization with acetylated tubulin. Scale bars: 10 μm.

To test for a role of SUMOylation in ciliary trafficking, we utilized a truncated form of SENP2 in which the first 72 amino acids are removed (ΔSENP2) (Benson et al., 2007; Hang and Dasso, 2002; Zhang et al., 2002). By truncating the N-terminus of SENP2, it is released from its normal, nuclear localization to the cytoplasm. Previous studies have therefore used overexpression of ΔSENP2 to dramatically change SUMO modification of all cytoplasmic proteins, as it is known to have high activity for cleaving all three SUMO isoforms (Benson et al., 2007). Interestingly, we found that the truncated version was enriched at the base of cilia in both IMCD3 cells (Fig. 3A) and MDCKII cells (data not shown), suggesting that it would be positioned to act on ciliary-targeted proteins. With this localization, it is possible that overexpression of ΔSENP2 could therefore deSUMOylate proteins that function or associate with primary cilia affecting the formation or maintenance of these cell organelles. To test whether global deSUMOylation altered cilia presence, cultured cells were transfected with GFP, GFP–SENP2 or GFP–ΔSENP2. Two different cell types were used to analyze these effects. MDCK cells were transfected, after ciliation had occurred and then analyzed 48 hours later. No significant difference was detected in the percentage of cells that possessed a primary cilium (Fig. 3B). As MDCK cells are grown so that they already possess a cilium at the time of transfection, this result indicates that altering the SUMOylation state of the cell does not affect cilia maintenance. To determine whether ciliogenesis is altered, IMCD3 cells were transfected with the same constructs, and then induced to form cilia by serum starvation. Under this paradigm, we also found no change in the percentage of ciliated cells indicating the SUMOylation is not crucial for the formation of cilia (Fig. 3C). To determine whether overexpression of ΔSENP2 had more subtle effects on cilia, we measured cilia lengths from IMCD3 cells. Compared to GFP-transduced cells, lengths of primary cilia transduced with GFP–ΔSENP2 were not significantly different (5.23±0.243 μm and 5.100±0.243 μm respectively).

To test whether SUMOylation is important for ciliary localization of AC3, we co-expressed AC3–GFP with ΔSENP2 in MDCK cells. When expressed with an empty vector, AC3–GFP was found in the cilium of over 90% of cells (*n*=37 cells) (Fig. 3D). In contrast, co-expression of AC3–GFP with ΔSENP2 severely reduced ciliary localization where only 7% of cilia (*n*=35 cells) were positive for AC3–GFP. As a control for specificity, we also tested ciliary localization of Arl13b. Co-expression of Arl13b–mCherry, with GFP–ΔSenp2 revealed that changing the SUMOylation state of the cell also did not affect its localization (Fig. 3E). This is consistent with a previous report showing that mutation of SUMO sites in Arl13b does not affect its localization (Li et al., 2012).

### Disruption of SUMOylation motifs inhibits ciliary localization of AC3

**Fig. 4.**
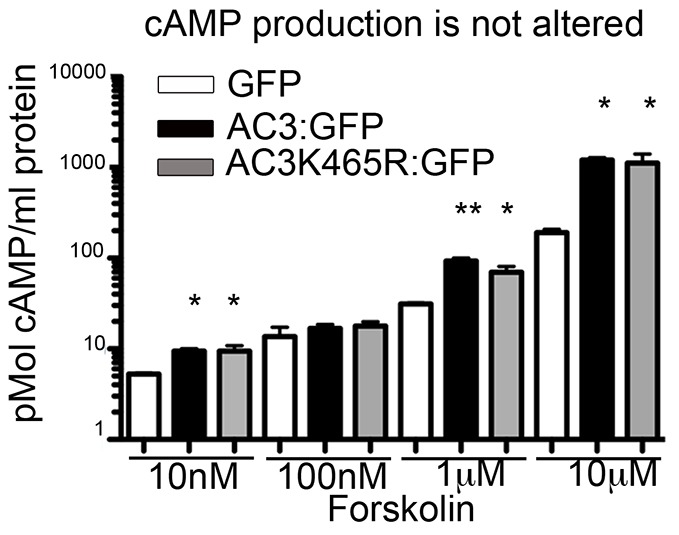
**AC3-K465R mutation does not alter function of AC3.** HEK293A cells were transfected with 1 µg of GFP, AC3–GFP (AC3:GFP) or AC3-K465R–GFP (AC3K465R:GFP). At 48 hours after transfection, cells were stimulated with forskolin for 10 minutes at the indicated concentrations, then collected to measure cAMP levels. Mutation of the SUMOylation site did not alter forskolin-stimulated cAMP production by AC3. Results are mean±s.e.m. (*n*=3 per treatment). *P<0.05; **P<0.005 (ANOVA).

As overexpression of ΔSENP2 effectively removes SUMO from all proteins, mislocalization of AC3 in the presence of ΔSENP2 does not address whether the targeting defect was directly due to altering the SUMOylation status of AC3. It could be that removal of SUMO from other proteins was responsible for the ciliary trafficking defects of AC3. To determine whether direct SUMOylation of AC3 is a crucial determinate of ciliary localization, we mutated its predicted SUMOylation site, K465, to an arginine residue. As AC3 is an integral membrane protein, we hypothesized that changes to membrane localization would alter cAMP production. To test for possible functional changes in cAMP, we assessed the function of AC3-K465R compared to wild-type AC3. HEK293 cells were transfected with the constructs and cAMP production in response to forskolin stimulation was measured. Cells expressing AC3-K465R produced similar levels of cAMP to those expressing wild-type AC3 (Fig. 4). These data provide an indication that the K465R mutation does not alter function of AC3, indicating proper localization to the surface membrane.

**Fig. 5.**
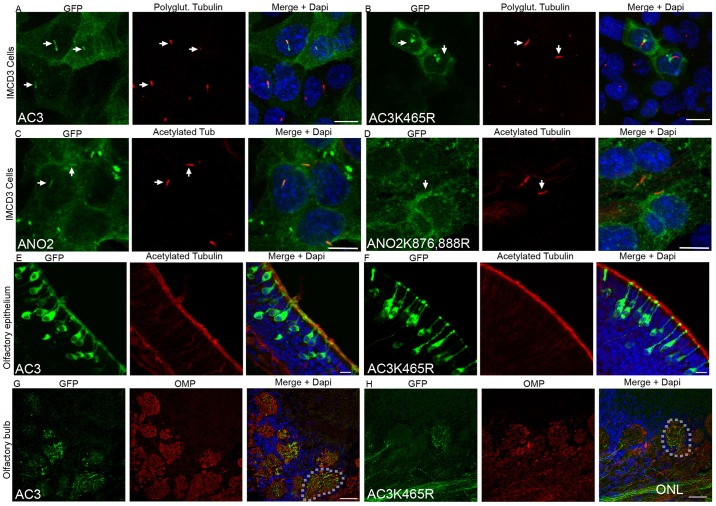
**SUMOylation sites are necessary for ciliary localization of transmembrane proteins.** (A,B) Site-directed mutagenesis of the SUMOylated lysine (K465) residue to arginine (R) prevents AC3–GFP from localizing to cilia (arrows). Wild-type AC3–GFP colocalizes with polyglutamylated tubulin, whereas AC3-K465R–GFP accumulates at the base of cilia. (C,D) Site-directed mutagenesis of both lysine residues to arginine (K876R and K888R) prevents ANO2–GFP from localizing to cilia (arrows). Wild-type ANO2–GFP colocalizes with acetylated α-tubulin (Acet. tub), whereas ANO2-K876,888R–GFP does not. (E,F) Coronal sections through the olfactory epithelium of mice transduced with either AC3–GFP or AC3-K465R–GFP. AC3–GFP colocalizes with acetylated α-tubulin (red) along the ciliary layer, whereas AC3-K465R–GFP is restricted to the dendritic knobs. (G,H) Both AC3–GFP and AC3-K465R–GFP localize to OSN axons as GFP-expressing axons can be seen innervating glomeruli (outlined) in the olfactory bulb. ONL, Olfactory nerve layer. Scale bars: 10 μm (A–F); 50 μm (G,H).

The ability of AC3-K465R to localize to primary cilia was then tested in cultured cells. IMCD3 cells were transduced with AC3–GFP or AC3-K465R–GFP adenovirus, and serum starved 24 hours later to induce ciliogenesis. Confocal imaging revealed that both the wild-type and mutant protein localized to the cell membrane. However, whereas AC3–GFP usually colocalized with the cilia marker polyglutamylated tubulin (64/74 cells), AC3-K465R–GFP was rarely seen in the primary cilium (9/90 cells) (Fig. 5A,B). Although these results indicate that the SUMO motif is necessary for AC3 localization, we sought to determine whether SUMOylation regulated ciliary trafficking of other transmembrane proteins. The Ca^2+^-activated Cl^−^ channel, ANO2 also localizes to cilia, and possesses two putative SUMO sites. To test the role of SUMOylation in ANO2 trafficking we mutated both lysine residues of the putative SUMOylation sites, at position 876 and 888, to arginine residues. Similar to AC3, the ablation of the putative SUMOylation sites in ANO2 also resulted in its loss of localization to the primary cilium (Fig. 5C,D).

The function of AC3 is perhaps best associated with its role in olfactory cilia, where it is necessary for the olfactory transduction pathway. Therefore, to test the role of SUMOylation in ciliary trafficking of AC3 *in vivo,* wild-type mice were intranasally injected with either AC3–GFP or AC3-K465R–GFP adenovirus constructs. Following a period of 10 days to allow virus expression, animals were killed, and the olfactory epithelium was section for immunofluorescence analysis. AC3:GFP signal was detected along the apical layer of the olfactory epithelium and colocalized with acetylated-tubulin (Fig. 5E), indicating ciliary localization. In contrast, whereas AC3-K465R–GFP localized to the cell membrane, in a similar manner to AC3–GFP, the fluorescence signal was not seen along the ciliary layer and instead only an intense fluorescence of the knob was seen (Fig. 5F), indicating that AC3-K465R–GFP is not enriched in the cilia. Analysis of OSN axons revealed that both constructs localized to the full length of axons along the olfactory nerve layer and in glomeruli in the olfactory bulb (Fig. 5G,H), providing further evidence that ablation of K465 does not affect membrane trafficking of AC3 in general.

**Fig. 6.**
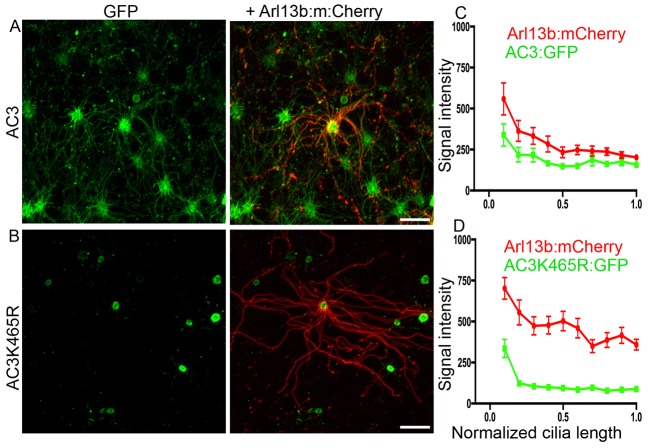
**Mutation of the SUMOylation site in AC3 site abolishes ciliary localization in live OSNs.** *En face* images of live OSNs transduced with either AC3–GFP (AC3:GFP) or AC3-K465R–GFP. To confirm to presence of cilia OSNs were co-transduced with Arl13b–mCherry (Arl13b:mCherry). (A) AC3–GFP colocalizes with Arl13b–mCherry along the full length of the cilia, whereas AC3-K465R (B) is restricted to the dendritic knob. Scale bars: 10 μm. (C,D) Quantification of signal intensity of the GFP from the base of the cilium to the tip. Results are mean±s.e.m. (*n*=8 cilia AC3, 13 cilia AC3-K465R).

To confirm that the loss of ciliary labeling was not due to fixation artifacts we used live *en face* imaging of OSNs transduced with either AC3–GFP or AC3K465R–GFP. As a means to visualize olfactory cilia and verify their presence, animals were also intranasally injected with an adenovirus expressing Arl13b–mCherry. In viewing the cilia *en face* as they extend from a dendritic knob, it is readily apparent that AC3–GFP and Arl13b–mCherry colocalized along the entire length of the cilium (Fig. 6A). Unlike AC3–GFP, AC3-K465R–GFP was restricted to dendritic knobs and was not apparent in cilia. The presence of Arl13b–mCherry-expressing cilia projecting from GFP-positive knobs demonstrates that these transduced OSNs possess cilia, but that AC3-K465R–GFP is not enriched in the cilia (Fig. 6B). Line-scan analysis of the fluorescent intensity of AC3–GFP compared to AC3-K465R–GFP confirmed a significant reduction in enrichment of AC3-K465R–GFP in olfactory cilia (Fig. 6C). Interestingly, the signal intensity for AC3-K465R–GFP is greater at the base of the cilium in both cultured cells and neurons compared to AC3–GFP, suggesting that because it is restricted from entering the cilia, it accumulates in this region of the cell (Fig. 6D). Arl13b–mCherry signals were comparable between animals. Taken together, these data suggest that ciliary localization of AC3 is directly dependent upon its ability to be SUMOylated.

### SUMOylation is not sufficient for ciliary localization

**Fig. 7.**
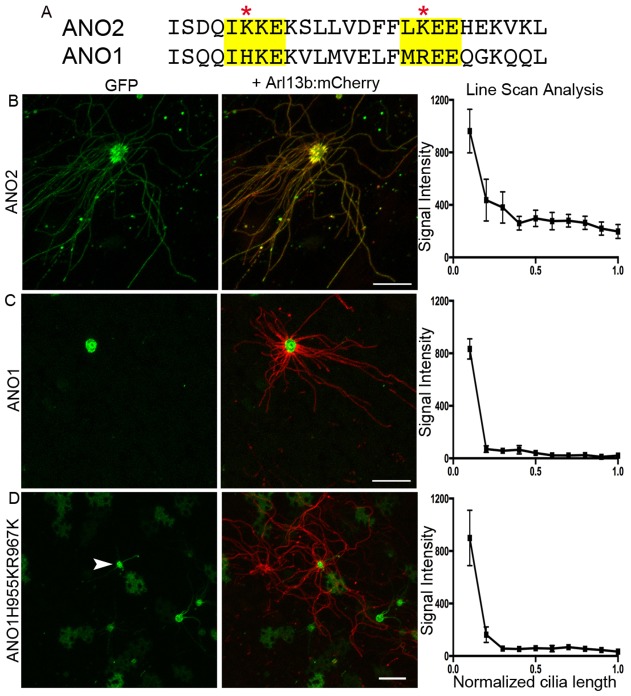
**SUMOylation sites are not sufficient for localization of ANO1 to olfactory cilia**. (A) Sequence alignment of the C-terminus of ANO2 and ANO1 reveals the SUMOylation motifs in ANO2 and conserved substitutions in ANO1. (B) *En face* images of live OSNs co-transduced with ANO2–GFP and Arl13b–mCherry reveal colocalization within the cilia. (C) Co-transduction of OSNs with ANO1–GFP and Arl13b–mCherry shows that ANO1 does not localize to cilia. (D) Mutation of H955 and R967 in ANO1 to lysine residues are not sufficient to permit ciliary entry of ANO1 as seen by lack of colocalization with Arl13b–mCherry in transduced OSNs. Quantitative results are mean±s.e.m. (*n*=10 cilia ANO2, 10 cilia ANO1, 11 cilia ANO1-H995KR967K). Scale bars: 10 μm.

Mutation of the putative SUMOylation site in AC3 demonstrates the necessity for these sequences but does not address whether SUMOylation is sufficient to permit entry of a transmembrane protein. However, bio-informatic analysis reveals that each of the ten type III adenylyl cyclase family members contains a putative SUMOylation motif. In addition, several are known to localize to cilia, meaning that this class of proteins is inappropriate to test for sufficiency. However, another olfactory transmembrane signaling protein, ANO2 (Billig et al., 2011; Stephan et al., 2009), contains two putative SUMO consensus motifs in the intracellular C-terminal tail. Fortuitously, ANO2 belongs to a gene family with a naturally occurring isoform, ANO1, which lacks these predicted SUMO motifs. Aligning the C-terminal tail of ANO1 and ANO2 reveals two homologous regions; however, in ANO1 the crucial lysine residues to which SUMO can conjugate are not present (Fig. 7A). In the nasal cavity, ANO1 localizes to the microvilli of vomeronasal sensory neurons and sustentacular cells, but is not detected in ciliated OSNs (Billig et al., 2011; Maurya and Menini, 2014). Therefore, ectopic expression of ANO1 is an ideal probe to test for the sufficiency of SUMOylation in targeting proteins to cilia. To address this question, we took advantage of the natural amino acid differences between ANO2 and ANO1 to ask when artificial reconstitution of SUMOylation sites would allow ciliary localization. To confirm that ANO2 and ANO1 localize differently to cilia we expressed GFP tagged wild-type constructs in cultured cells and native OSNs. Although ANO2–GFP colocalization was detected with ciliary markers, ANO1–GFP was not detected in the cilia of OSNs (Fig. 7A,B) nor in those of MDCK or IMCD3 cells (supplementary material Fig. S2A,B). Using site-directed mutagenesis we added the crucial lysine residues in ANO1 to reconstitute the two putative SUMOylation sites both individually and in combination. By using dual injection with Arl13b–mCherry, we analyzed ciliary localization of the GFP-tagged proteins *en face* in live OSNs. Although removal of SUMOylation sites had a clear effect on ciliary localization of AC3 *in vivo*, the addition of putative SUMOylation sites to ANO1 did not increase its ciliary enrichment (Fig. 7C, supplementary material Fig. S2C,D). Line-scan analysis of signal intensity further confirmed the lack of ANO1 variants in the cilia of transduced OSNs (Fig. 7). Thus, SUMOylation might be necessary for ciliary enrichment of some proteins, but it is not sufficient to promote localization of an protein that is otherwise excluded from cilia.

## DISCUSSION

Primary cilia are crucial cellular organelles that on many cells act as biological antennae detecting extracellular signals. Their importance is underscored by the genetic disorders, termed ciliopathies, associated with dysfunctional cilia. Although some of these disorders are caused by mutations altering cilia structure or presence, many are associated with mutations affecting function or ciliary localization of signal transduction proteins. In this way, mutations targeting specific amino acid sequences that are necessary for specific trafficking mechanisms could cause the crucial proteins to fail to localize to cilia, leading to disease phenotypes. How signaling proteins are targeted to and trafficked into cilia is therefore key to understanding the mechanisms and potential treatments of ciliopathy disorders. The results of the studies presented here provide insight into the requirements for trafficking transmembrane proteins into the cilium, and demonstrates a direct functional role for SUMO modification in ciliary trafficking in an *in vivo* system.

Although SUMOylation has been most heavily studied in the context of nuclear import and transcription regulation, recent studies have demonstrated roles for SUMOylation, both direct and indirect, in the function and trafficking of transmembrane proteins, particularly in neurons (Luo et al., 2013). In the synapse, SUMOylation of the kainate receptor GluK2 (also known as GRIK2) is needed for induced internalization (Martin et al., 2007). Furthermore SUMOylation of K^+^ channels at the membrane is able to alter pore properties. Indirect roles for trafficking of transmembrane proteins have also been identified. For example, SUMOylation of collapsing response mediator protein 2 (CRMP2, also known as DPYSL2) is necessary for surface localization of the Na^+^ channel Nav1.7 (Dustrude et al., 2013). Similarly, an indirect role for SUMOylation has been postulated in the trafficking of specific transmembrane proteins into the cilia of *C. elegans* (Li et al., 2012). In that work, Arl13b was shown to harbor SUMOylation sites, although mutation of these sites did not affect its localization to cilia. In Arl13b-null worms, the transmembrane proteins PKD-2 and ODR-10 are mistrafficked, showing aberrant localization in dendrites. Whereas expression of wild-type Arl13b can correct this defect, the SUMO mutant Arl13b is unable to do so. These studies demonstrate a role for SUMOylation of Arl13b in the complete ciliary localization of PKD-2 and ODR-10. However, as both were still present in the cilium, these experiments do not demonstrate a requirement for SUMOylation in ciliary entry. Here, we have shown that direct SUMOylation of AC3 is a crucial determinant of its ciliary enrichment. Immunoprecipitation studies show that AC3 is a SUMO substrate and that mutation of the SUMO site blocks SUMO3 binding. Thus, the loss of AC3 localization when the SUMO site is mutated can be assumed to be due to the loss of SUMOylation. That AC3-K465R still localizes to the OSN axons and produces cAMP in response to forskolin stimulation indicates that SUMOylation of AC3 does not affect global processes, but rather might be specific for aiding ciliary entry.

Our work demonstrates a new role for a protein modification process in the regulation of specific ciliary proteins. Ciliary enrichment is a complicated process for which multiple mechanisms have been identified that often act in concert for the trafficking of individual proteins (Berbari et al., 2008a; Bhogaraju et al., 2013; Geng et al., 2006; Hurd et al., 2010; Hurd et al., 2011; Jenkins et al., 2006; Jenkins et al., 2009; Kee et al., 2012; McEwen et al., 2007; Sharma et al., 2008). Additionally, these mechanisms often regulate a subset of ciliary-targeted proteins but are not ubiquitous in their requirement. Furthermore, individual proteins in specific classes are not necessarily regulated by the same mechanisms. For example, ciliary localization of some GPCRs is driven by a motif found in the third intracellular loop; however, this motif is largely absent from olfactory receptors, which are ciliary localized (Berbari et al., 2008a). Sequence analysis of other proteins known to be enriched in olfactory cilia suggests that SUMOylation might regulate ciliary localization of some, but not all of these proteins. In addition to ANO2, SUMO motifs are present in CNGB1b, CNGA2 and GNG1. However, SUMOylation motifs are not present in several other proteins that localize to olfactory cilia, including olfactory marker protein (OMP), GNAL, GNB1, CNGA4 and five olfactory receptors that were analyzed. Our data suggests that disruption or mutations in SUMOylation pathways within a cell could alter the localization of ciliary proteins. Additionally, mutations in SUMOylation motifs in signaling proteins targeted to the cilium could underlie disease phenotypes due to changes in trafficking.

An interesting phenomenon in the localization of proteins to cilia is that several mechanisms have been identified that are necessary but not sufficient for ciliary entry. For this study, we were able to take advantage of a naturally occurring difference between two highly homologous proteins. Although the majority of the SUMOylation sequence is present in ANO1, both of its potential motifs lack the crucial lysine residues that are present in ANO2. Interestingly, ANO1 and ANO2 show differences in their localization patterns in the nasal cavity. ANO2, is expressed in OSNs and localizes to cilia, whereas ANO1 is found in sustentacular cells and vomeronasal sensory neurons, both of which possess microvilli (Billig et al., 2011). The lack of SUMO sites in ANO1, paired with its lack of ciliary localization allowed us to address whether these motifs are sufficient for entry. Reconstitution of the SUMO motifs, both individually and combined, however, was unable to turn ANO1 into a ciliary-targeted protein. This finding is not without precedent, as several other trafficking mechanisms have also been found to be necessary but not sufficient for ciliary targeting. For example, trafficking of CNGA2 in cilia requires interaction with CNGB1b, which possesses an RVxP motif. Insertion of this motif into CNGA2 is insufficient for targeting it to the cilium in the absence of CNGB1b. The lack of ciliary localization and SUMO sites in mammalian ANO1 is interesting considering a recent report showing that *C. elegans* ANO1 localizes to sensory cilia (Wang et al., 2013). Analysis of ANOH-1, the *C. elegans* homolog, reveals an intracellular SUMO motif in the N-terminus of the protein. The presence of this SUMO site, in conjunction with other unidentified mechanisms could explain the difference in ciliary localization of *C. elegans* and mammalian ANO1.

The loss of AC3, but not Arl13b, targeting to cilia due to changes in SUMOylation state suggests that membrane interactions of ciliary-targeted proteins could contribute to how SUMO affects localization. It is possible that SUMOylation is important for ciliary localization of transmembrane but not cytoplasmic proteins, particularly given that cytoplasmic proteins can vary greatly in the localization mechanisms used. This idea is supported by recent analysis of how the transcription factor Gli2 localizes to cilia. Several mechanisms known to regulate ciliary trafficking of transmembrane proteins, such as co-factor interaction, nuclear import signals and phosphorylation are dispensable for Gli2 ciliary localization (Santos and Reiter, 2014). Additionally, Santos and Reiter found that mutation of the Gli2 SUMOylation motif did not affect ciliary enrichment of the protein. Interestingly, the Gli2 SUMOylation mutants display increased transcriptional activity, raising the possibility that SUMO modification might be important for limiting Gli2 in the absence of activation and maintaining it in the cilium. Santos and Reiter also reported being unable to detect components of the SUMO pathway at or near the primary cilia of cultured cells. This is in contrast to our findings for SUMO3, which was associated with cilia, and that of another study, which found GFP-tagged Ubc9 in the cilia of *C. elegans* (Li et al., 2012). Thus, it is likely that the use of tagged ectopically expressed proteins in our study aided in the identification of SUMO pathway proteins associated with the cilium. The difference in results also raises the possibility that endogenous SUMO and SENP proteins are difficult to detect, possibly requiring specific fixation or antigen retrieval techniques to reveal their localization. These findings provide further evidence that that the diversity of mechanisms used for cilia localization varies, likely due to the membrane interactions of individual proteins and the cell type they are found in.

The identification that SUMOylation regulates protein entry into the cilium adds to growing evidence of similarities and shared mechanisms regulating nuclear and ciliary trafficking. Previous studies have identified other similarities including the use of RAN gradients, importin interactions, nuclear localization signals, size exclusion and the presence of nucleoporin proteins (NUPs) at the cilia base (Dishinger et al., 2010; Fan et al., 2011; Hurd et al., 2011; Kee et al., 2012; Kee and Verhey, 2013). An important role for NUPs is regulating SUMOylation of proteins trafficking through the nuclear pore. Some NUPs, such as Nup358 are recognized SUMOP E3 ligases (Pichler et al., 2002), whereas others help to keep SENP proteins at the nuclear pore complex. The finding of NUPs at the base of cilia provides further support for SUMOylation being a mechanism that regulates cilia entry. More recently, another SUMO E3 ligase, TOPORS, was found to localize to be the base of the connecting cilium in photoreceptors (Chakarova et al., 2011; Weger et al., 2003). The identification of SUMOylation as a regulator of ciliary trafficking adds an additional link between nuclear and ciliary similarities.

In summary, our results demonstrate a significant role for SUMOylation in the ciliary localization of select olfactory signaling proteins. Chronic overexpression of the SUMO protease SENP2 prevented the ciliary localization of AC3. This effect is specific for trafficking mechanisms because overexpression of SENP2 did not affect the presence or length of cilia. In agreement with this, we found that mutation of the SUMOylation site in AC3 blocked ciliary entry in both cultured cells and *in vivo* in OSNs. The mutation not only blocked localization, but also appeared to cause an accumulation of protein at the ciliary base. In addition localization of AC3-K465R in the axons of OSNs was unaffected. Thus AC3 trafficking to the membrane in general is not altered by mutation of the SUMO site, but entry into the cilium is inhibited. These results indicate that the SUMO modification is needed for passing through the ciliary base. That SUMO itself was not found accumulated in the cilium indicates that it might be cleaved from modified proteins and rapidly removed upon moving through the ciliary pore. Whether SUMOylation affects trafficking of other transmembrane proteins into cilia remains to be tested. Interestingly, initial screens of odorant receptors did not identify putative SUMO sites leaving the mechanisms regulating the trafficking of these important proteins still largely unknown. Our results clearly link SUMO modification of signaling proteins to ciliary entry and highlight a role for SUMOylation in the subcellular trafficking of proteins in OSNs.

## MATERIALS AND METHODS

### Bio-informatics

Sequence analysis was performed using SUMOplotsp2.0. The following sequences were used for analysis (unless specified all sequences are from *Mus musculus*); AC1, NP_033752.1; AC2, NP_705762.2; AC3, NP_612178.2, NP_004027.2 (*Homo sapiens*), NP_001156014.1 (*Danio rerio*), XP_003641104.2 (*Gallus gallus*), XP_008116786.1 (*Anolis carolinensis*), NP_001096154.1 (*Xenopus tropicalis*); AC4; NP_536683.1; AC5, NP_001012783.3; AC6, NP_031431.2; AC7, NP_001032812.2; AC8, NP_033753.2; AC9, NP_033754.2; AC10, NP_766617.2; CNGA2, NP_031750.2; CNGA4, NP_001028489.1; CNGB1, NP_001182342.1; Gnal, NP_796111.2; Gng13, NP_071867.1; Gnb1, NP_001153488.1; ANO2, NP_705817.2; Olfr15, NP_032788.2,; Olfr2, NP_035113.1; Olfr151, NP_997547.1; Olfr73, NP_473431.1; Olfr78, NP_001161975.1; ANO1, NP_848757.4.

### Constructs and adenovirus preparation

Plasmids containing cDNA inserts used in this study were generously provided as follows: SENP2 and SUMO3 (Jorge Iniguez-Lluhi, University of Michigan, Ann Arbor, MI), AC3 (Randall R. Reed, Johns Hopkins University, Baltimore, MD), Arl13b (Tamara Caspary, Emory, Atlanta, GA), ANO2 and ANO1 (Haiqing Zhao, Johns Hopkins University, Baltimore, MD). Disruption of SUMOylation sites were performed using a Strategene site-directed mutagenesis kit.

For expression in native tissue, recombinant GFP- and mCherry-fused cDNAs were cloned into the vector p-ENTR by TOPO cloning methods. The inserts were then recombined into the adenoviral vector pAD/V5/-dest using LR Recombinase II (Life Technologies, Carlsbad CA). Viral plasmids were digested with PacI and transfected into HEK293 cells. Following an initial amplification, a crude viral lysate was produced, and used to infect confluent 60-mm dishes of HEK293 cells for amplification according to the ViraPower protocol (Life Technologies). Adenovirus was isolated with the Virapur Adenovirus mini purification Virakit (Virapur, San Diego, CA), dialyzed in 2.5% glycerol, 25 mM NaCl and 20 mM Tris-HCl, pH 8.0, and stored at −80°C until use.

### Immunoprecipitations

For immunoprecipitation from native olfactory epithelium, CD-1 mice were deeply anesthetized with CO_2_, and decapitated. All animal experiments were performed according to approved guidelines (see section on intranasal injections). Olfactory epithelium was dissected into in immunoprecipitation buffer containing 10 mM Tris-HCl, pH 7.5, 1 mM EDTA, 150 mM NaCl and protease inhibitors with 1% Nonidet P-40. Samples with NEM contained 20 mM NEM. The tissue was then homogenized on ice and rocked for 1 hour on a nutator at 4°C in 1 ml of immunoprecipitation buffer. The lysate was then centrifuged at 1000 ***g*** to pellet nuclei for 5 minutes. Supernatant was then rocked with 100 µl of protein A agarose beads for 1 hour, centrifuged at 10,000 ***g*** to clear beads and then incubated with 2 µl of rabbit anti-AC3 antibody (Santa Cruz Biotechnology, sc-588) overnight at 4°C. For immunoprecipitations from cells, at 48 hours post-transfection, HEK293 cells were lysed on ice for 30 minutes in 500 µl immunoprecipitation buffer. After douncing, cells were centrifuged at 1000 ***g*** for 5 minutes and supernatant was incubated overnight at 4°C with antibody-conjugated beads. 5% of the supernatant was used for starting material. Beads were washed three times for 5 minu with immunoprecipitation buffer with 1% Triton X-100 and once with immunoprecipitation buffer without Triton X-100. Supernatant was analyzed by SDS-PAGE as described previously (Jenkins et al., 2006; Jenkins et al., 2009).

### Cell culture

Madin Darby canine kidney (MDCKII) cells were grown in Dulbecco's modified Eagle's medium (DMEM) supplemented with 10% fetal bovine serum and 1% penicillin, streptomycin and Gluta-MAX I. MDCK cells were grown 7 days post-confluence on transwell filter supports (0.4 mm pore size, Corning, Tewksbury, MA) prior to fixation. IMCD3 cells were grown in DMEM with F12 supplemented with 10% fetal bovine serum (FBS) and 1% penicillin-streptomycin. To induce cilia formation, cells were serum starved for 48 hours post-transfection. For the cAMP assay, HEK293 cells were transfected with 1.5 mg of either AC3–GFP for AC3K465R–GFP plasmid. At 48 hours following transfection, cells were stimulated with forskolin diluted in DMSO. Cells were incubated at 37°C for 10 minutes then collected, and cAMP levels were assayed following the manufacturer’s instructions (Cayman Chemical, Ann Arbor, MI). Measurements were made on triplicate samples from three separate experiments.

For immunostaining, cells were fixed with 4% PFA, quenched with 50 mM NH_4_Cl in PBS, permeabilized with 0.1% Triton X-100 in PBS, and blocked with 0.3% goat serum (Life Technologies) in PBS. Samples were incubated in primary antibodies for 1 hour at room temperature, washed with 1×PBS, incubated with fluorophore-conjugated secondary antibodies for 1 hour, washed with 1×PBS, incubated with DAPI and mounted using Prolong Gold (Life Technologies). Monoclonal antibody against acetylated α-tubulin (T6793, Sigma-Aldrich) and secondary antibodies were used at 1:1000 dilutions. For γ-tubulin (T3559, Sigma-Aldrich) immunostaining, cells were fixed with a 50:50 mix of methanol and acetone at −20°C for 20 minutes. Cells were then washed with PBS and treated as previously described. *z*-series were captured on an Olympus Fluoview 500 confocal microscope equipped with a 405-nm laser diode with a 430–460 nm bandpass filter, a 488-nm laser with a 505–525 nm bandpass filter, a 543-nm laser with a 560-nm long-pass filter, and a 633-nm laser with a 660-nm long-pass filter. 60×1.40 numerical aperture (N.A.) oil objectives were used. The fluorescence signals of compressed *z*-stacks were quantified using NIH ImageJ software.

### Intranasal injections

All animals were handled according to the guidelines for animal care and procedures approved by the University Committee for the Use and Care of Animals at the University of Michigan and the Institutional Animal Care and Use committee at the University of Florida. Neonatal animals were intranasally injected with 20 µl of adenovirus at ages P7–P9. Animals were held in the supine position and plastic tubing was inserted into the nasal opening for virus delivery. Animals were held in this position for 3–5 minutes following injection. Animals were then returned to their cages for 10 days to allow expression of the ectopic protein(s).

### Tissue preparation

For immunostaining, mice were deeply anesthetized and then cardiac perfused with 4% paraformaldehyde (PFA). The lower jaw, teeth and hair were removed from snouts prior to post fixation in 4% PFA for overnight at 4°C. Snouts were then decalcified in 0.5M EDTA in 1×PBS overnight, then cryoprotected in 10%, 20% and 30% sucrose for 1 hour, 1 hour and overnight, respectively, at 4°C, and embedded in OCT compound (Tissuetek). Coronal cryosections were sliced at a thickness of 10–12 µm and mounted onto Superfrost Plus slides (Fisher Scientific). Immunostaining was carried out as above.

For confocal *en face* imaging of transduced OSNs, animals were anesthetized with CO_2_, rapidly decapitated, split along the cranial midline, and the olfactory epithelium was dissected out in PBS. The removed olfactory turbinates were placed with the apical (ciliary) surface against a glass coverslip and held with a tissue slice holder (Warner Instruments, Hamden, CT). Turbinates were bathed in 1×PBS for imaging on a Nikon Ti eclipse-A1R confocal microscope with a 60×1.40 N.A oil objective.

For imaging of tissue sections, confocal imaging was performed as described previously (Jenkins et al., 2006; McIntyre et al., 2012). Exposures were adjusted so that the maximal pixel intensities were at least half saturation. For adenoviral olfactory epithelium experiments, in order to minimize contribution of ciliary protein localization from uninfected OSNs, we used compressed *z*-stacks corresponding only to the thickness of the infected OSN. Images were analyzed with ImageJ software (NIH, Bethesda, MD), and statistics were performed with Prism 5 software from Graphpad Prism Software (San Diego, CA). Adjustments of contrast and brightness were performed using Adobe Photoshop CS5.1 (San Jose, CA).

## Supplementary Material

Supplementary Material
