# Supplementary material for: SUMOylation regulates ciliary localization of olfactory signaling proteins
Source: J Cell Sci. 2015 May 15;128(10):1934–45. doi: 10.1242/jcs.164673 (PMC4457158; doi:10.1242/jcs.164673)
Supplement: Supplementary Material [file supp_128_10_1934__index.html]

SUMOylation regulates ciliary localization of olfactory signaling proteins — Supplementary Material 

# SUMOylation regulates ciliary localization of olfactory signaling proteins

## JCS164673 Supplementary Material

**Files in this Data Supplement:**

- **Supplementary Material**
