# Supplementary material for: SUMOylation regulates ciliary localization of olfactory signaling proteins
Source: J Cell Sci. 2015 May 15;128(10):1934–45. doi: 10.1242/jcs.164673 (PMC4457158; doi:10.1242/jcs.164673)
Supplement: Supplementary Material [file supp_128.10.1934_JCS164673.pdf]

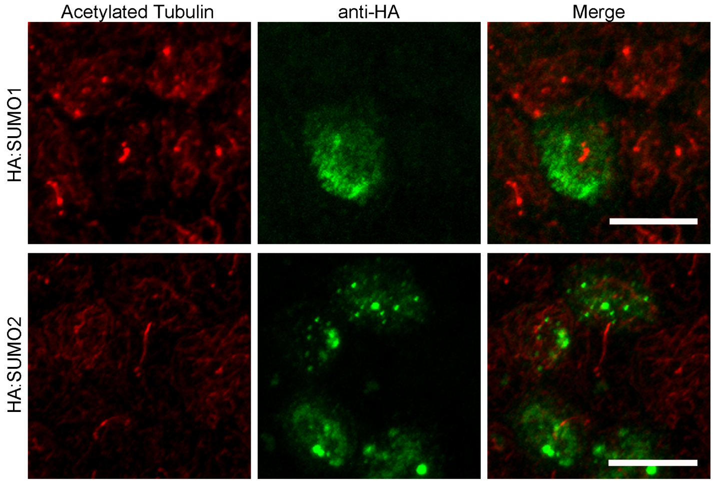

Supplementary Figure 1: Localization of SUMO1 and SUMO2 in MDCK cells. MDCKs transfected with HA:SUMO1 or HA:SUMO2, and immunostained with antibodies to HA and acetylated α-tubulin. Accumulation of these SUMO isoforms is not seen at the base of the cilia. Scale bar = 10μm

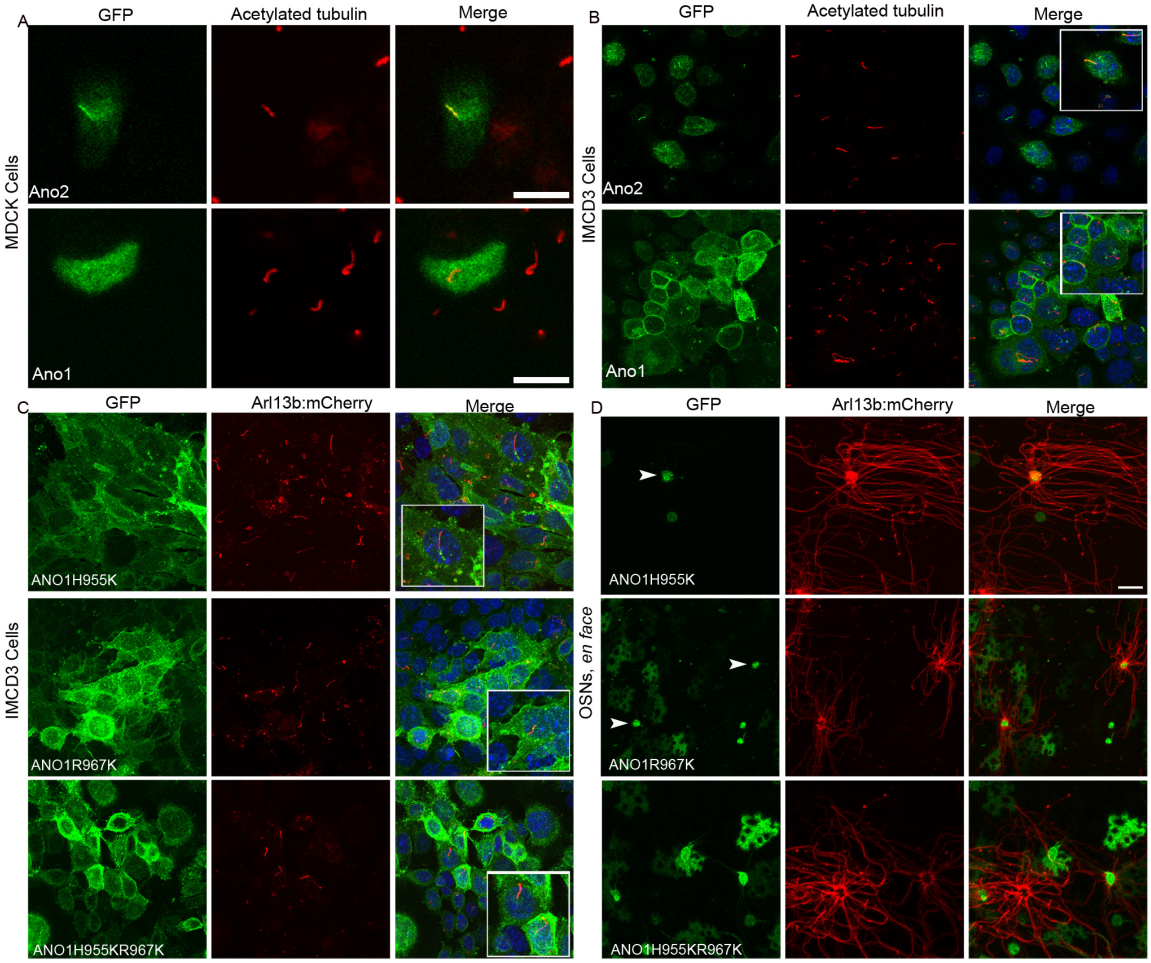

Supplementary Figure 2: Localization differences between ANO2 and ANO1. (A, B) ANO2:GFP co-localizes with acetylated tubulin in MDCK (A) and IMCD3 (B) cells, while ANO1:GFP does not. (C) Reconstitution of SUMO sites in ANO1, either individual or together, was not sufficient for ciliary localization in IMCD3 cells. (D) SUMO sites were not sufficient for cilia localization in olfactory cilia. Scale bars = 10μm
